# How does emotional content affect lexical processing?

**DOI:** 10.1080/02699931.2013.851068

**Published:** 2013-11-08

**Authors:** David Vinson, Marta Ponari, Gabriella Vigliocco

**Affiliations:** Department of Cognitive, Perceptual & Brain Sciences, University College London, London, UK

**Keywords:** Valence, Word recognition, Lexical decision

## Abstract

Even single words in isolation can evoke emotional reactions, but the mechanisms by which emotion is involved in automatic lexical processing are unclear. Previous studies using extremely similar materials and methods have yielded apparently incompatible patterns of results. In much previous work, however, words' emotional content is entangled with other non-emotional characteristics such as frequency of occurrence, familiarity and age of acquisition, all of which have potential consequences for lexical processing themselves. In the present study, the authors compare different models of emotion using the British Lexicon Project, a large-scale freely available lexical decision database. After controlling for the potentially confounding effects of non-emotional variables, a variety of statistical approaches revealed that emotional words, whether positive or negative, are processed faster than neutral words. This effect appears to be categorical rather than graded; is not modulated by emotional arousal; and is not limited to words explicitly referring to emotions. The authors suggest that emotional connotations facilitate processing due to the grounding of words' meanings in emotional experience.

Language is a powerful vehicle for the expression of emotion, and in influencing the emotional states of others. Even single words in isolation can evoke strong emotional reactions, such as the feelings associated with strongly positive or negative words like *kitten* or *murder* even though they do not directly refer to emotions themselves. Emotional content even plays a role in automatic lexical processing of single words, as indicated by reliable effects of emotional valence in lexical decision, a relatively shallow task in which words must be distinguished from non-words (e.g., Estes & Adelman, 2008a, 2008b; Kousta, Vigliocco, Vinson, Andrews, & Del Campo, 2011; Kousta, Vinson, & Vigliocco, 2009; Larsen, Mercer, Balota, & Strube, 2008). While all of these studies provide support for the involvement of words' emotional characteristics in their processing, the precise mechanisms involved still remain entirely unclear. This is because different studies of lexical processing have found different and apparently incompatible results even when the same task (e.g., lexical decision) is used.

An essential first step in investigating emotional content of words is to take into consideration other non-emotional characteristics of words that may also affect their processing. This issue was highlighted by Larsen, Mercer, and Balota (2006): in a meta-analysis of emotional Stroop studies, they showed that previously reported effects of emotional valence (i.e., numeric ratings indicating the extent to which a word is positive, neutral or negative) can change dramatically once confounding variables such as length, frequency and orthographic neighbourhood size are taken into account. However, even after controlling for these non-emotional variables, results of lexical decision studies remain in conflict. Estes and Adelman (2008a, 2008b) and Larsen et al. (2008) reported slower lexical decision reaction times (RTs) for negative than positive words. This has been interpreted in terms of attentional vigilance: heightened and/or extended attention to negative stimuli (e.g., Pratto & John, 1991), which would slow any decision (such as lexical decisions) on other aspects of the stimuli. In contrast, Kousta et al. (2009) found a processing advantage for both negative and positive over neutral words, which they explain in terms of greater motivational relevance of emotionally loaded stimuli (e.g., Lang, Bradley, & Cuthbert, 1997). Kousta et al. argued that this discrepancy in findings may have arisen from a relative lack of neutral words in the data-sets tested previously or due to the lack of control of additional potentially confounding variables that also affect lexical decision latencies, such as ratings of familiarity and age of acquisition (AoA). In addition, Larsen et al. (2008) found that the effect of valence was modulated by the arousal of words such that a negative disadvantage was present for medium-low arousing words, but no effect was observed for highly arousing negative words. Estes and Adelman (2008a) argued instead for a far more constrained role of arousal, and Kousta et al. (2009) argued against the involvement of arousal (although Kousta et al. did not explicitly test valence × arousal interactions).

All of these previous studies were conducted using lexical decision data from a single source: the English Lexicon Project (ELP, Balota et al., 2007), so in addition to questions about the different assumptions and approaches taken by previous authors, one may also wonder about the extent to which the findings may be related to quirks of that particular item set. Here, we take advantage of an entirely independently obtained large-scale set of lexical decision data (British Lexicon Project [BLP]; Keuleers, Lacey, Rastle, & Brysbaert, 2012), to try and resolve these questions. Our analyses compare models based on different *a priori* theoretical assumptions concerning the role of valence in word processing, controlling non-emotional variables known to affect lexical decision RTs. We begin by fitting baseline models in which all the non-emotional predictors mentioned above are taken into account, then add specific terms embedding different assumptions about the role of valence, pitting them against each other to test the theoretical accounts of emotion effects in lexical processing. The main question addressed by these contrasts concerns whether negative words show a disadvantage relative to other word types (e.g., Estes & Adelman, 2008b) or whether emotionally valenced words are advantaged relative to neutral words (e.g., Kousta et al., 2009). We also compare models in which the effects of valence are treated categorically (as in Estes & Adelman, 2008b) to those where it is treated as a continuous measure (as in Kousta et al., 2009). This is important because quadratic effects in continuous, non-linear models (like the quadratic valence model favoured by Kousta et al.) do not necessarily imply symmetry between positive and negative (e.g., the maximum may not occur precisely at the midpoint of the valence scale). However, if a continuous non-linear measure does not outperform its categorical counterpart in which symmetry is enforced, we have no evidence for an imbalance between positive and negative words. After assessing how well different measures of valence perform after taking baseline variables into account, we move on to evaluating the role of other aspects of emotional content besides just valence, assessing the extent to which valence effects may instead be explained or modulated in terms of arousal.

Finally, we test whether the effects of emotional valence differ for words specifically referring to emotions (e.g., fear, love, shame) versus words that are only valenced (e.g., prison, justice, cheat). So far, large-scale studies of emotion in lexical processing have not addressed the question of whether the valence effect is being driven by a specific, limited set of words: those referring explicitly to emotion, or whether it generalises to all valenced words. For example, Altarriba and Bauer (2004) argue that emotion words are sufficiently different to other types of words that we ought to consider words as falling into three categories: concrete, abstract and emotion words, thus predicting valence effects to be limited to emotion words (see also Moseley, Carota, Hauk, Mohr, & Pulvermüller, 2012, who argue that emotion words are embodied in the physical manifestations of experiencing emotion such as facial expression and posture). Instead, Kousta et al. (2009, 2011) argue that emotion provides a mechanism to ground all words in internal states, thus predicting that valence effects should be general across the vocabulary.

## METHOD

### Data

The BLP data-set comes from an extremely large-scale lexical decision study including 28,730 words, in which each participant performed more than 28,000 lexical decision trials (half the set of words, plus an equal number of non-words) over the course of multiple sessions totalling approximately 16 hours (Keuleers et al., 2012). From the full set of words in the BLP, we selected those 1374 words for which valence ratings were available from the Affective Norms for English Words (ANEW) (Bradley & Lang, 1999), or from the additional ratings described in Kousta et al. (2009, 2011). Next, we filtered out those words for which BLP participants were extremely inaccurate: those with overall accuracy less than 67% in the BLP (*n* = 56, e.g., *larkspur, dryad, godhead*). This is an important step as widely unfamiliar words are likely to elicit slow RTs and to receive neutral valence ratings from participants. Finally, we removed five words for which concreteness and imageability ratings were not available, leaving 1313 words for analysis. Of these, 856 were in common with the set from the ELP that Kousta et al. (2009) analysed.

#### Measures of emotional valence

We centred the scale of the original valence ratings which ranged from 1 to 9, so as to range from −4 (most negative) to +4 (most positive) with 0 reflecting neutrality. We then created the following measures embedding different theoretical assumptions concerning valence. The most essential distinction concerns the direction of valence effects in order to differentiate accounts of emotion processing. Accounts based on attentional vigilance would predict a disadvantage for negative words, while motivational accounts would instead predict an advantage for emotional words (whether positive or negative) over neutral. In addition, we compare models in which valence is considered as a continuous measure, versus models in which it is discretised, as a test of previous claims that effects of emotion should be considered all-or-nothing (e.g., Estes & Adelman, 2008a, 2008b).

#### Continuous valence

These measures treat valence as a continuous value, varying from most negative (−4) through neutral (0) to most positive (+4).

##### Linear

Linear measure includes only the linear relationship between valence and RT. If negative words are slower than other words (e.g., Estes & Adelman, 2008a, 2008b; Larsen et al., 2008), we expect to find a negative slope (RTs decrease with increasing valence).

##### Polynomial

Polynomial measure includes linear and quadratic components of valence.^1^ If valenced words are faster than neutral words with no difference between positive and negative (e.g., Kousta et al., 2009), we expect a negative quadratic coefficient while the linear coefficient would offer no further benefit.

#### Discrete valence

These measures treat valence as categorical rather than continuous but they embed the same basic contrast as above.

##### Negative/positive

Negative/positive measure includes two discrete valence classes: negative (valence < 0) and positive (valence ≥ 0) valence levels. If negative words are slower than other words, these two categories should differ. This model is the simplest discrete counterpart to the linear measure above and was preferred by Estes and Adelman (2008b) as more complex measures they tested did not account for the data any better than a simple categorical model.

##### Valenced/neutral

Valenced/neutral measure treats positive and negative as a single class, compared to neutral (emotional: |valence| > 1.5; neutral: |valence| ≤ 1.5). If emotional words are faster than neutral words, we expect to find differences between these two categories (just as we would for the quadratic term of the polynomial measure).

### Design and analysis

We fit a variety of hierarchical regression models described in more detail below, in each case testing for a partial effect of valence on lexical decision latencies, using any of the four proposed valence measures. We conducted our analyses on log-transformed RT (excluding error trials), first fitting models to trial-level data and then to item averages.^2^

Analysis of trial-level data was carried out using linear mixed-effects models (packages lme4: Bates & Maechler, 2009; and languageR: Baayen, 2009) in the R programming environment (R Core Team, 2013). Model fits included random intercepts for both subjects and items, as well as random slopes by subjects (for emotional predictors only, which are constant for each item). Analysis of item averages was carried out using ordinary least squares regression.

In all of the analyses we conduct upon valence measures, we always begin with a baseline model including the following non-emotional factors that were controlled in all the previous studies we have mentioned: number of letters; log(HAL frequency), orthographic neighbourhood size (all from Balota et al., 2007); we also included additional non-emotional predictors which Kousta et al. (2009) argued to be essential in order to unambiguously interpret effects as emotional in nature: mean positional bigram frequency (from Balota et al., 2007); ratings of concreteness, imageability and familiarity (from Coltheart, 1981) and AoA ratings (from Stadthagen-Gonzalez & Davis, 2006). For each of these measures, we included polynomial transformations (up to third order)^3^ and retained them in the baseline model only if they were significant predictors. As a result, our tests of the partial effects of emotional variables provide results that can be unambiguously attributed to emotion rather than other characteristics of words with which emotional properties may be confounded.

### The role of arousal

Some previous studies have shown that effects of valence are modulated by arousal (Estes & Adelman, 2008a; Larsen et al., 2008, but see Kousta et al., 2009). Using a similar modelling approach as above, we test the role of arousal in two ways. First, we consider arousal as a categorical measure (high arousal words vs. low arousal words), testing valence × arousal interactions for any of the valence measures described previously which turn out to be significant predictors of lexical decision RT. If arousal modulates the effect of valence, we should see such an interaction. Second, we treat arousal as a control variable, testing in a different set of models whether unique effects of valence can be observed after variation related to arousal is taken into account. This is particularly important for models distinguishing valenced from neutral words (i.e., quadratic term of the polynomial measure and valenced/neutral measure) as valenced words exhibit a strong tendency to be more arousing as well (Bradley & Lang, 2000).

### Emotion words versus emotionally valenced words

To address this issue, we used Wordnet-Affect (Strapparava & Valitutti, 2004) to identify emotion words. Wordnet-Affect classifies words according to their organisation in Wordnet. Any word with an emotional sense is considered “emotional,” thus this is a conservative classification. We hand-classified a few additional words as potentially emotional (e.g., courage, craven, stern) ending up with 193 of the 1313 words classified as emotion words. To test whether emotion words alone are responsible for valence effects, we fit models as above, testing for interactions between valence and emotion-word classification. If emotion words drive the effects observed, we should see an interaction such that the valence effects are restricted to emotion words (or at least, should differ between emotion and non-emotion words).

## RESULTS

### Fitting baseline models

It is no surprise that many of the non-emotional variables were significant predictors of lexical decision latencies, consistent with a wealth of previous studies. For the purposes of the present study, we simply note here that higher-order polynomial transformations offered significant improvement in performance over linear-alone components for several of the predictors. Moreover, although some factors were not significant predictors in the baseline model (i.e., concreteness, imageability and summed positional bigram frequency), we retained them as (linear) predictors^4^ along with the following predictors that were significant in the (reduced) baseline model: third-order polynomial transformations: (log frequency, number of letters, number of orthographic neighbours, familiarity); linear terms (AoA). Plots of the significant predictors are provided as Supplemental Figure 1, and parameter estimates for baseline models are provided as Supplemental Table 1.

**Figure 1.**
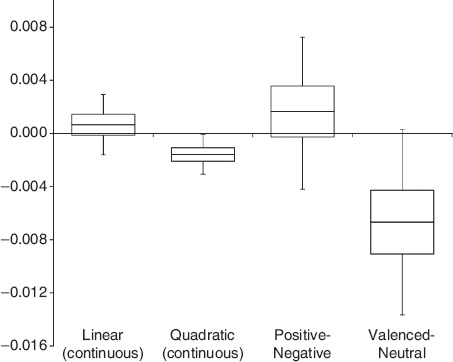
Graphical depiction of parameter estimates of the different valence predictors. Continuous measures: estimate of the slope (linear measure) and quadratic coefficient (polynomial measure), log(RT) scale. Categorical measures: estimate of the difference between the two conditions. Horizontal line = mean parameter estimate. Box depicts 50% confidence interval of the parameter estimate; whiskers depict 95% confidence interval.

**Table 1.** Partial effects of the different valence measures (trial-level analyses), after taking non-emotional variables into account. Parameter estimates come from different models, each of which contains all of the baseline variables (see Figure 1) along with a single measure of valence. Dependent measure: log(RT)

*Valence measure*	*Estimate (Std err.)*	t *statistic*
Linear	.00066 (.00161)	.57
Polynomial		
(Linear term)	.00052 (.00116)	.45
(Quadratic)	−.00158 (.00076)	−2.07
Negative/positive	.00166 (.00286)	.58
Valenced/neutral	−.00670 (.00357)	−1.87

### Measures of emotional valence

We tested the effects of valence by adding each of the valence measures described above to the best-fit baseline model above, thus always allowing us to evaluate the partial effect of valence only after non-emotional variables were taken into account. We also added that same valence term in each model as a random slope by subjects (in analysis of trial-level data) as doing so provided significantly better fit than models with only subject and item intercepts.

Only those measures in which valenced words differ from neutral words were reliable predictors of lexical decision RT. Neither the linear continuous measure nor the discrete measure contrasting negative and positive predicted RT once confounding factors were taken into account (see Figure 1 and Table 1). To assess whether the continuous (quadratic) measure offers sufficient additional explanatory power beyond the simplest categorical measure contrasting valenced to neutral words, we fit one additional set of models, in which we entered second-order polynomial valence along with the categorical measure, and we compared the models using likelihood ratio tests. There was no significant improvement gained by adding this additional term (log-likelihood ratio for valenced/neutral model = 7624.1; log-likelihood ratio for combined model = 7630.0; χ^2^(9) = 11.877, *p* = .220) with comparable results for analysis of item averages.^5^

At this stage, the data suggest that the effect of valence is best described as a simple, categorical contrast between words with emotional associations and those without. Thus, when non-emotional variables are taken into account, we see that a categorical measure of valence, regardless of polarity, is sufficient to account for emotional effects in word processing.

### The role of arousal

Here, we focus upon those valence measures that were reliable predictors in the previous section (i.e., second-order Polynomial and Valenced/Neutral), assessing whether they can be accounted for, or modulated, by arousal.

First, we tested the interaction between arousal and each of the two valence measures (continuous and categorical). For these analyses, we discretised arousal, using a median split to characterise words as low or high arousal (contrast coded). For trial-level analyses, we included both main effects and the interaction as random slopes by subjects. We found that the main effect of valence persisted, with no effect of arousal category and no interaction between the two: quadratic coefficient estimate = −.00338 (*SE* = .00110), *t* = −3.067, arousal main effect and interaction |*t*| < 1.2; categorical coefficient estimate = −.0135 (*SE* = .0050), *t* = −2.725, arousal main effect and interaction |*t*| < 1 (analyses of item means found no effect of arousal nor interactions with valence).

Next, we added a continuous measure of arousal into the models, testing whether a partial effect of a valence measure could still be seen after arousal was taken into account. For trial-level analysis, this meant including random slopes by subject for arousal as well as for valence. We started by adding arousal to the baseline model described above. When arousal was the only emotional variable included, its effects were significant (estimate of the slope = −.0050 (*SE* = −.0020, *t* = −2.518): more arousing words elicited faster responses. We then added a valence measure to this baseline + arousal model. For both the polynomial and the categorical valence measure, effects persisted once arousal was taken into account: quadratic coefficient estimate = −.00261 (*SE* = .00144), *t* = −2.627; categorical coefficient estimate = −.0112 (*SE* = .0046), *t* = −2.410), with the partial effect of arousal not reaching significance (|*t*| < 1). These findings were replicated in analyses of item averages. These effects of emotion can thus be attributed to valence rather than arousal.

### Emotion words versus emotionally valenced words

As in the second set of analyses considering the role of arousal, we tested whether the effects of valence described above were different for emotion words and those not referring to emotional states (using Wordnet-Affect, Strapparava & Valitutti, 2004) by testing for statistical interactions.

Just like our analyses involving arousal, the main effect of valence was unchanged, with no effect of Wordnet-Affect category and no interaction. For trial-level analysis: quadratic coefficient estimate = −.00197 (*SE* = .00085), *t* = −2.31, Wordnet-Affect category main effect and interaction |*t*| < 1; categorical coefficient estimate = −.00969 (*SE* = .00419), *t* = −2.31, Wordnet-Affect category main effect and interaction |*t*| < 1.02. Again, analyses of item averages showed the same pattern. It appears that these effects of valence are not simply the consequence of words specifically referring to emotions, but are more general.

## DISCUSSION

Our analyses show a reliable, consistent and rather simple pattern of emotion effects in lexical processing: once potentially confounding variables are taken into account, lexical decisions to emotionally valenced words are recognised faster than those to neutral words. This finding differs from some previous studies (Estes & Adelman, 2008a, 2008b; Larsen et al., 2008): those investigating ELP data, using a more limited set of words (from ANEW, Bradley & Lang, 1999) and crucially, for which some important control variables are unavailable. Those studies also conducted analysis over item averages only, allowing the possibility that valence effects observed there may have been magnified or distorted as a consequence of treating these values as point estimates rather than varying by subjects. However, the present results suggest that was not the case: here, we observed no difference in the patterns of results whether conducting analysis on item averages or upon trial-level data. Our results also appear to differ from those reported by Kousta et al. (2009) although consistent with their overall conclusions. We found no benefit in considering valence as a continuous measure: the second-order polynomial valence model is no better than the simplest categorical model (valenced vs. neutral). As it turns out, the present study and Kousta et al. actually yield the same conclusions: we reanalysed their data using a categorical model (valenced vs. neutral) and found that their continuous model was no better than the simplest categorical version of it.

We also found this categorical effect of valence was not modulated by arousal: once confounding variables are taken into account, arousal and valence did not interact, and even when we regressed out variance related to arousal first, categorical valence was still a significant predictor. This finding resonates with recent neuroimaging evidence using a highly controlled set of words, in which activation in rostral anterior cingulate cortex (an area associated with emotion processing) is modulated by valence (regardless of whether it is positive or negative) and not by arousal (Vigliocco et al., 2013). Finally, the effect does not seem to be limited to words explicitly referring to emotional states (Altarriba & Bauer, 2004) but seems to be more general in nature. At first glance, this seems to be contrary to a prediction derived from Moseley et al. (2012) that words explictly referring to emotions would specifically benefit from body-specific activation related to physical expression of the emotional states themselves. However, Havas and Matheson (2013) have proposed an embodied theory in which bodily states (particularly facial expression) rapidly and automatically evoked by emotional content are deeply linked to language processing. If so, this would apply more generally to emotional valence rather than being restricted to words explicitly referring to emotional experience.

One important limitation that needs to be addressed is the relatively small magnitude of the emotion effects we report here, which may otherwise go unnoticed. Our estimate of the difference between valenced and neutral words (–.0067 in log(RT) units) only corresponds to approximately 4 ms difference at the median RT observed in the source data-set (529 ms). Similarly, a quadratic coefficient of −.00158 corresponds to a difference of about 6 ms between the most extremely valenced words and the most neutral ones. This is substantially smaller than the valence effects reported in other studies, the most comparable being Kousta et al. (2009). Their analysis of data from the ELP revealed a valence advantage around 15 ms, and their experiment using a smaller set of highly controlled words yielded a 24 ms emotion advantage. The substantial reduction in the emotion effect we observed cannot just be attributed to our analysis of trial-level data as the item analysis revealed a similarly small effect; this reduction of the valence effect may simply be a consequence of practice effects in the large-scale lexicon projects (see Keuleers et al.; Figure 1). After all, participants in the BLP data-set performed more than 28,000 lexical decisions, compared to approximately 3400 for ELP participants and only 240 in Kousta et al. (2009) so it is no surprise that the magnitude of this effect appears reduced. Most important, however, is that emotion effects persist even after participants have experienced thousands of lexical decision trials: emotional content is sufficient to facilitate lexical decisions even in highly practiced participants.

Why would emotional content facilitate lexical processing? Under general motivational accounts of processing (Lang et al., 1997), both negatively and positively valenced items are relevant to survival and well-being albeit for different reasons. Crucial in this regard is the involvement of emotion processing systems even for lexical stimuli which do not exhibit obvious low-level visual characteristics argued to be evolutionarily linked to positive or negative emotions (vs. emotional expressions or visual properties of dangerous entities). In various recent proposals, the involvement of emotional systems has been argued to provide a means for grounding abstract concepts in internal experience, whether through internal experience of emotional states (Kousta et al., 2011; Vigliocco, Meteyard, Andrews, & Kousta, 2009), feedback from facial expression and/or other bodily correlates of emotion expression (Havas & Matheson, 2013; Moseley et al., 2012), or the interaction of approach and avoidance systems as indexed by measures of danger and usefulness (e.g., Wurm, 2007). Regardless of the specific theoretical account, however, emotional systems appear to be involved even when single words are processed in isolation.

### Supplementary material

Supplementary Figure 1 and Table 1 are available via the “Supplementary” tab on the article's online page (http://10.1080/02699931.2013.851068.2013.851068).

### Supplemental data

The item list is archived in the ESRC Data Store (oai:store.ac.uk:archive:1079), as are the valence norms from Kousta et al. (2011) that were used in the present study.

## Notes

1 Kousta et al (2009) used restricted cubic splines to model nonlinearity in valence, finding that such a model outperformed one with linear valence alone; here we report a more commonplace measure including linear and quadratic terms as these relate directly to the theoretical alternatives described in previous literature. We also tested models based on restricted cubic splines; they perform comparably to the polynomial models described above.

2 Some previous studies of this nature only report analyses on average response times for single words, averaged across multiple subjects but treated as point estimates (e.g., Estes & Adelman, 2008a, 2008b; Kousta, Vinson, & Vigliocco, 2009; Larsen et al., 2008). Such approaches may overestimate the quality of any predictor as an essential component of variability has been discarded. In the present study, we conduct analyses of trial-level data (nearly 50,000 observations) as well as item averages, allowing us to test whether emotional variables still play a role when individual variability is taken into account. The British Lexicon Project is particularly appropriate for such an approach as each participant saw a very large number of words compared to any other data set.

3 The same results obtain throughout if non-linearity in the non-emotional variables is modelled using restricted cubic splines rather than polynomial transformations.

4 Non-significant predictors were kept in the baseline model in case their absence may have altered the effects of emotional valence in subsequent models.

5 The same patterns were also observed when the same type of modelling approach was taken, but using untransformed RT rather than log-transformations as we report here. The patterns also did not change substantively if participant slopes were left out of the models (i.e., fitting only random intercepts for subjects and items, although see Barr, Levy, Scheepers & Tily, 2013 for arguments against such an approach).
